# Research on Quantitative Evaluation of Remote Sensing and Statistics Based on Wireless Sensors and Farmland Soil Nutrient Variability

**DOI:** 10.1155/2022/3646264

**Published:** 2022-01-19

**Authors:** Weishuai Ji, Yaqiu Liu

**Affiliations:** ^1^College of Information Science and Engineering, Shandong Agricultural and Engineering University, Jinan, Shandong 251100, China; ^2^College of Resources and Environment, Shandong Agricultural University, Taian, Shandong 271018, China

## Abstract

The combination of wireless sensor networks and radio technology can form a new type of communication network. The emergence of wireless sensor networks has effectively solved the problems existing in radio technology, but traditional wireless sensor networks and radio technology networks cannot be directly applied to wireless sensors. On this basis, this paper studies the remote sensing of soil nutrient variability in agricultural land using wireless sensors. Due to traditional farmland management and agricultural systems, farmland soil nutrient variability has led to polarization: fertile soil has excess nutrients, reducing the use rate of chemical fertilizers and polluting high-quality farmland. Traditional farming methods can no longer meet the requirements, and modern technology must be used to comprehensively understand the spatiotemporal variability of soil nutrients during plant growth. Remote sensing technology has the advantages of accuracy, speed, economy, and regular monitoring. It provides new ideas and technical guarantees for soil quality evaluation in land development and consolidation projects. This paper also studies the use of statistical quantitative evaluation technology to carry out multidimensional statistical quantification of soil protection function evaluation at a given location. Finally, wireless sensor networks are used to analyze the relationship between several natural factors and quantitative estimation of soil protection. Based on wireless sensor technology, this paper studies the variability of farmland soil nutrients and statistical quantitative evaluation, hoping to lay a foundation for the development of agriculture and statistics.

## 1. Introduction

In the past few decades, wireless sensor networks have received great attention from academia and industry. Sensor nodes communicate over a short distance through a wireless network and communicate with each other to perform tasks. For soil detection, hundreds or even thousands of sensor nodes can be used to collect data [1]. Due to the low cost of sensor nodes, constant changes in availability, and limited sensor energy, the development of routing protocols suitable for soil detection, extending the life of the network, and increasing the utilization rate are of great significance to the promotion of agricultural development [2]. On this basis, this article investigates the variability of farmland soil nutrients. China still mainly adopts traditional farmland management measures; that is, the same fertilization measures are adopted instead of considering the spatial distribution of farmland soil nutrient variability, which results in fertility [3]. The oversupply of nutrients in the plots caused unnecessary waste, while also reducing fertilizer utilization and causing large-scale agricultural nonpoint source pollution [4]. On the barren land, the supply of nutrients was limited and could not meet the growth needs of crops [5]. In view of the complex soil types and extensive soil information, traditional farmland management methods can no longer meet the requirements [6]. To achieve effective fertilization and production management, it is necessary to use modern science and technology to understand the variable characteristics of farmland nutrients. The successful application of remote sensing technology in soil organic matter monitoring, soil moisture monitoring, vegetation index, and other aspects has brought new ideas and technical guarantees for soil evaluation [7]. According to the quality of development and land consolidation and remote sensing based on the variability of soil nutrients in farmland, this paper also conducts research on statistical quantitative evaluation [8]. China continues to implement land development and consolidation projects and carries out acceptance evaluation of real estate development and consolidation projects [9]. Quantitative assessment of soil quality after completion becomes more and more important. At present, it is necessary to strengthen the research on soil quality evaluation after land consolidation. On the one hand, soil quality evaluation requires a lot of manpower, material resources, and financial resources [10]. On the other hand, the standards for soil quality evaluation are not standardized, so this work is rarely carried out [11]. At present, the evaluation of development and land consolidation mainly adopts artificial field analysis and statistical quantitative evaluation methods [12]. This kind of evaluation method cannot be comprehensively evaluated, and it is time-consuming, laborious, and costly and lacks objective comparison and the macroscopic nature of evaluation [13]. The above research can contribute to further in-depth research on quantitative science and remote sensing technology [14]. It can also enrich China's land development and consolidation theories, accelerate the development of land development theoretical systems and the adoption and evaluation of consolidation, and promote the development of precision agriculture and digital agriculture [15].

## 2. Related Work

The literature proposes that the application of remote sensing technology to wireless sensors creates a wireless sensor network, which overcomes the limitations of traditional wireless sensor networks. The sensor node evaluates the conditions independently or in cooperation with other nodes and uses the allowed channel to complete data transmission [16]. If nutrient activity is detected in the main user's soil, the remote sensing technology immediately leaves the current channel [17]. The literature states that agricultural land is an extremely precious natural resource, with a slow origin time, a small amount, and uneven spatial distribution [18]. From a natural point of view, its formation is affected by the five elements of parent material, biology, climate, topography, and time [19]. These five factors have their own characteristics and properties, but they collectively control the formation of soil development and characteristics from different angles [20]. The literature discusses the scheme of combining wireless sensor network technology and remote sensing technology for environmental geological monitoring of mining areas, and in view of the problems and difficulties faced by the scheme, it proposes the use of these two technologies to establish a dynamic model of vegetation net initial value productivity (NPP) in the mining area. The literature shows that the soil protection module of the remote sensing technology model conducts quantitative research on soil protection in a certain area, so that its spatial attributes are visible in three dimensions, and uses soil-based detectors to reveal the relationship between soil protection and various natural functions [21]. The literature introduces the research on the temporal and spatial variability of nutrients in the soil, which is an urgent problem to be solved in agronomy, forestry, horticulture, ecology, and other disciplines [22]. Research has also developed from qualitative research to quantitative research and expanded from the original classical statistical methods to quantitative methods. Compared with classical statistical research, variables should be purely random and statistical research should involve regional variables. The literature shows that the spatial variability of soil nutrients has a large-scale effect, and soil sampling at different scales demonstrates the corresponding spatial structure characteristics and changing laws. The spatial variability of the same trait varies greatly at different scales, and large-scale studies will examine the structural characteristics of small-scale soils. In order to accurately and systematically record soil variability, soil scholars gradually try to study the variability of soil nutrients from different perspectives.

## 3. Principles of Wireless Sensors and Remote Sensing Technology

### 3.1. Wireless Sensor

Wireless sensor networks use independent sensor nodes and wireless technology to communicate with other nodes to relay information to appropriate destinations. After the sensor node processes the data through its internal processor, it sends the integrated data to the next hop node. Research on wireless sensor networks has been conducted for more than 30 years and is rapidly expanding and improving to meet the needs of military applications.

**Figure 1:**
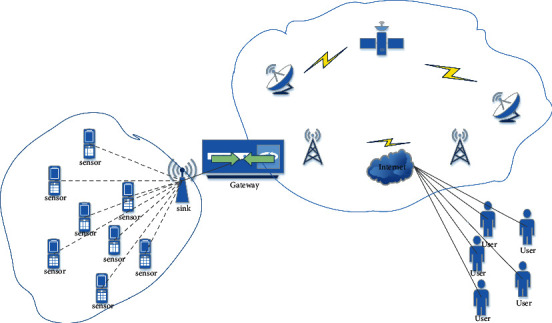
WSN topology.

The basic idea of WSN is that the capacity of each individual sensor node is limited, but the overall performance of the entire network is sufficient to perform the required tasks. The network topology of traditional WSN is shown in Figure 1.

Some characteristics of wireless sensor networks are as follows: First, compared with some deterministic networks, the nodes of sensor networks are used randomly. Second, wireless sensor networks are designed for specific scenarios, so the types and numbers of sensors equipped in different scenarios are also different. For example, building monitoring requires only a small number of sensors, which can be placed separately.

### 3.2. Remote Sensing Image Processing

#### 3.2.1. Geometric Precision Correction of Images

The point selection technology provided by image registration, that is, geometric correction, includes “(image to image)” and “(image to reference map).” In this experiment, the ground control point coordinates are measured on the spot and the examples are the first two methods of selecting points.

The polynomial correction model is as follows:

(1) $$\begin{array}{c} x=F_{x}\left(u,v\right)=\sum_{i=0}^{n}\sum_{j=0}^{n-i}a_{ij}u^{i}v^{j}, \end{array}$$

where *a*_*ij*_ and *b*_*ij*_ are undefined coefficients, *n* is the polynomial order, and *F*_*x*_ and *F*_*y*_ are thedistortion correction function.

#### 3.2.2. Jurisdiction Calibration

Radiometric calibration refers to quantifying the relationship between the brightness value in the corresponding field of view of the remote sensing sensor and the digital quantitative output. Therefore, the following relationship exists between the received radiation intensity and its data value:

(2) $$\begin{array}{c} L_{\lambda }=\left(\frac{{\text{LMAX}}_{\lambda }-{\text{LMIN}}_{\lambda }}{Q_{\text{cal}}\max}\right)Q_{\text{cal}}+{\text{LMIN}}_{\lambda }, \end{array}$$

where *L*_*λ*_ is the radiation intensity received by the sensor (W/(m^2^·*μ*m·sr)), Qc_al_ is the gray value of the pixel, and Q_calmax_ is the maximum DN value.

Spectral reflectivity *r* outside the atmosphere at each band is as follows:

(3) $$\begin{array}{c} r=\frac{\pi {\text{Ld}}^{2}}{E\cos\left(\theta \right)}. \end{array}$$

Here, *E* is the average spectral irradiance outside the atmosphere of the corresponding area (unit: W·m^−2^·*μ*m^−1^·sr^−1^), *d* is the astronomical unit of the distance between the sun and the Earth, and *ɵ* is the zenith angle of the sun (unit: Rad).

Atmospheric external albedo *α*_toa_ is as follows:

(4) $$\begin{array}{c} a_{\text{toa}}=\sum c_{i}r_{i}\;\left(i=1,2,3,4,5,6,7\right). \end{array}$$

Here, *c*_*i*_ is the weight coefficient of the *i*-th band.

Ground albedo *a* is as follows:

(5) $$\begin{array}{c} a=\frac{a_{\text{toa}}-a_{\text{path}}}{\tau^{2}\text{sw}}. \end{array}$$

Here, sw is the one-way transmission of the atmosphere and *a*_path_ is the radiation range (value between 0.025 and 0.04).

#### 3.2.3. Correction of Radiation Errors Caused by the Sun's Altitude

Generally, the following relationship can be used to solve the radiation error correction caused by the sun's altitude:

(6) $$\begin{array}{c} f\left(x,y\right)=\frac{g\left(x,y\right)}{\sin\theta }. \end{array}$$

In this equation, *ɵ* is the angle of the sun's height, *g*(*x*, *y*) is the pixel coordinate of the sun's image at the elevation angle, and *f* (*x*, *y*) is the pixel coordinate of the image obtained under direct sunlight.

#### 3.2.4. Atmospheric Correction

The goal of atmospheric correction is to eliminate these effects caused by the atmosphere and light in remote sensing images and to obtain true reflectance data of ground objects.

(7) $$\begin{array}{c} L_{\lambda }=\frac{X_{\lambda }}{A}+B, \end{array}$$

(8) $$\begin{array}{c} P_{\text{TOA}}=\frac{\pi L_{\lambda }d^{2}}{{\text{ESUN}}_{\lambda }\cos\left(\theta_{s}\right)}, \end{array}$$

where *X*_*λ*_ is the DN value of each band, parameter *A* is the absolute calibration gain of the image product after radiation correction, *L*_*λ*_ is the brightness of each band, *d* is the astronomical distance between the sun and the Earth, parameter *B* is the calibration offset of the image product, *θ*_*s*_ is the zenith angle of the sun, and ESUN is the average solar illuminance of the outer atmosphere at wavelength *λ*.

## 4. Statistical Quantitative Evaluation Analysis and Application Research on the Variability of Farmland Soil Nutrients

### 4.1. Principles of the Quantitative Evaluation of Soil Quality

The InVEST model is a model for evaluating ecosystem service functions. It includes three main modules and several submodules of terrestrial, freshwater, and marine ecosystems. The results of the work are visually presented in the form of a map, which helps to better understand the nature of ecosystem services. The difference between erosion and actual erosion reflects the reduction in potential erosion in each block. The latter means that the product of the amount of sand and the degree of sediment retention indicates the ability of the grid block to intercept the sediment or other sediments on the slope. The USLE equation ignores this important hydrological process. It is calculated as follows:

(9) $$\begin{array}{c} {\text{SEDRET}}_{x}={\text{RKLS}}_{x}-\text{USLE }x+{\text{SEDR}}_{x}, \end{array}$$

(10) $$\begin{array}{c} \text{PKLS}x=Rx\cdot Kx\cdot \text{LS}x, \end{array}$$

(11) $$\begin{array}{c} \text{USLE }x=Rx\cdot Kx\cdot \text{LS}x\cdot Cx\cdot Px, \end{array}$$

(12) $$\begin{array}{c} \text{SDRE}x=\text{SE}x\sum_{y=0}^{x-1}, \end{array}$$

where SE*x* is the sediment retention efficiency in the *x* grid, SEDRET*x* and SEDR*x* are the soil retention and sediment retention in the *x* grid, and SE*z* is the sediment retention efficiency in the *z* grid. RKLS*x* is the potential soil loss in grid *x* based on terrain and climatic conditions, and USLE*x* and USLE*y* are the actual erosion values of grid *x* and its upstream grid *y*.

Geographic detectors are divided into four types: factor, ecology, risk, and interaction. The factor detector is designed to detect the spatial differentiation of the dependent variable and the degree of interpretation of the spatial differentiation *Y*, expressed by the *q* value, and its expression is as follows:

(13) $$\begin{array}{c} q=1-\frac{\sum_{a=1}^{B}M_{nc_{d}^{2}}}{M_{c^{2}}}=1-\frac{\text{DDE}}{\text{DDR}}, \end{array}$$

(14) $$\begin{array}{c} \text{DDE}=\sum_{a=1}^{B}M_{nc_{d}^{2}}, \end{array}$$

(15) $$\begin{array}{c} \text{DDR}=M_{C^{2}}. \end{array}$$

Here, *a* = 1,…, *B* is the division of variable *Y* or factor *X* and DDE and DDR are the sum of the intralayer variance and the total variance of the entire region, respectively.

Ecological detector: it is used to judge whether there are significant differences in the influence of two factors, *b*_1_ and *b*_2_, on the spatial distribution of *Y* attributes, which is characterized by the *S* statistic:

(16) $$\begin{array}{c} S=\frac{A_{x1}\left(A_{x2}-1\right){\text{DDE}}_{x1}}{A_{x2}\left(A_{x1}-1\right){\text{DDE}}_{x2}}, \end{array}$$

(17) $$\begin{array}{c} {\text{DDE}}_{x1}=\sum_{a=1}^{B1}M_{nc_{d}^{2}}, \end{array}$$

(18) $$\begin{array}{c} {\text{DDE}}_{x2}=\sum_{a=1}^{B2}M_{nc_{d}^{2}}, \end{array}$$

where AXO and An represent the sample sizes of factors *X*_1_ and *X*_2_, respectively, and DDE*n*1 and DDE2 are the sum of the intralayer variances of the layer formed by *X*_1_ and *X*_2_, respectively. The null hypothesis *H*_0_ is DDE*n* = DDE_*X*2_. If H_0_ deviates from the significance level *c*, this means that *X*_1_ and *X*_2_ are significantly different in the spatial distribution of *Y*.

Risk detector: it is used to determine whether there is a significant difference in the attribute mean of the two subregions and uses the *t* statistic to test

(19) $$\begin{array}{c} t_{{\bar{T}}_{g=1}-{\bar{T}}_{g=2}}=\frac{{\bar{T}}_{g=1}-{\bar{T}}_{g=2}}{{\left[\text{Csv}\left({\bar{T}}_{g=1}\right)/m_{g=1}+\text{Csv}\left({\bar{T}}_{g=2}\right)/m_{g=2}\right]}^{1/2}}, \end{array}$$

where *T*_*e*_ is the average value of the attributes in the subdomain *g*, *m* is the number of samples in the subrange *g*, and Csv is the variance.

### 4.2. Soil Data Processing

#### 4.2.1. Rainfall Erosivity *R* Factor

The greater the precipitation erosion coefficient, the greater the intensity of soil erosion. The methods to obtain this coefficient are listed:

(1) The classic algorithm is as follows:
  (20) $$\begin{array}{c} R=E\times I_{30}, \end{array}$$
where *R* is the erosion activity of rain, *E* is the total kinetic energy of rain, and I_30_ is the rainfall intensity within 30 minutes.
This model has extremely high requirements for data sources and must be based on a large amount of precipitation data. It has limitations in the complexity of obtaining kinetic energy and precipitation rate data, and its relatively narrow scope limits further applications.
The modified formula for the calculated *R* factor formula is as follows:
  (21) $$\begin{array}{c} R=\sum_{i=1}^{12}\left[1.735\times 10^{\left(1.5\;\mathrm{lg}Ti^{2}/T-0.8188\right)}\right]\times 17.02, \end{array}$$
where *T* is the annual average rainfall (mm) and Ti is the monthly rainfall (mm).
(2) We established a power index structure daily rainfall erosivity model:
  (22) $$\begin{array}{c} \text{Rds}=R\sum_{i=1}^{k}{\left(\text{Ti}\right)}^{\lambda }. \end{array}$$
*k* represents the number of days, Ti represents the precipitation of the *i*-th day, Ti ≥ 12 mm is required; otherwise, the value is 0, Ty12 is the annual average rainfall with a daily rainfall ≥12 mm, and Td12 is the daily average rainfall with a daily rainfall ≥12 mm.
(3) We established the national daily replacement model (half-month rainfall erosivity model):
  (23) $$\begin{array}{c} \text{Mi}=\alpha \sum_{j=1}^{k}{\left(\text{Dj}\right)}^{\beta }. \end{array}$$

Here, Mi is the erosion activity value in the *i*-th half-month period (MJ·mm·hm^−2^·h^−1^), *α* and *β* are the model parameters, *k* represents the number of days in a half-month, and Dj represents the *j-*th day of the first half-month. The daily precipitation requires precipitation ≥12 mm.

The numbers *α* and *β* represent model parameters reflecting the characteristics of precipitation in the area. According to the daily precipitation data, the values of *α* and *β* in the station are estimated according to

(24) $$\begin{array}{c} \beta =0.8363+\frac{18.144}{P\left(d12\right)}+\frac{24.455}{P\left(y12\right)}, \end{array}$$

(25) $$\begin{array}{c} \propto =21.586\beta^{-7.1891}. \end{array}$$

This method uses long-term continuous daily rainfall as the main unit of data. It is generally considered to be the most accurate and most representative method of the actual rainfall erosion rate, and long-term continuous data are required.

According to the applicability of the model, combined with the existing meteorological and precipitation data, the final method of calculating the *R* factor is as follows:

(26) $$\begin{array}{c} R=\sum_{i=1}^{12}73.989\times {\left(\frac{P_{i}^{2}}{P_{a}}\right)}^{0.7387}. \end{array}$$

Here, *R* is the erosion activity value (MJ·mm·hm^−2^·h^−1^), *P*_*i*_ is the precipitation of the *i*-th month (mm), and *P*_*a*_ is the average annual precipitation (mm).

The latitude and longitude of a certain area and its 6 surrounding counties and cities and the *R* value of precipitation erosion are shown in Table 1.

This paper uses the monthly precipitation data of a certain region and 6 counties and cities over 35 years from 1986 to 2020 to calculate the annual value of precipitation erosivity and the value of the annual average rainfall erosivity factor.

**Figure 2:**
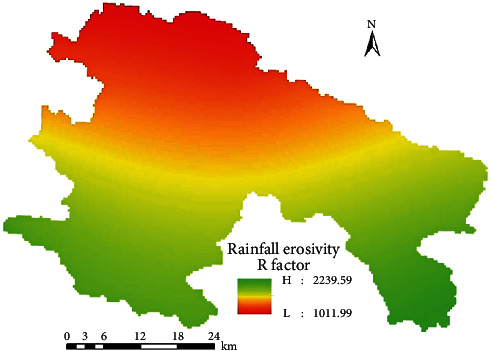
Spatial distribution of the *R* factor of rainfall erosivity.

On this basis, the Kriging interpolation method was used on the GIS platform to generate an *R* layer based on the 6 counties from 1986 to 2020, and then, the mask was extracted to obtain the spatial distribution of the *R* factor of the A district and counties, as shown in Figure 2.

Figure 2 shows that the *R* value gradually decreases from south to north. There are high-value areas adjacent to the southwest and southeast of area *A* and high-precipitation areas *D* and *E*; a low value area appears in the dry hot area adjacent to the northwest of the valley.

#### 4.2.2. Soil Erodibility *K* Factor

The soil erodibility *K* factor reflects the difficulty of separating and transporting soil with different particle sizes, and the difference in the *K* factor reflects the sensitivity of different types of soil to erosion. The *K* value can be obtained by querying data, actual measurement, and calculation. The most common method for querying data is the Vischmeier nomogram. This method requires more data on soil structure coefficients and permeability levels. At present, China's soil data are mainly based on the results of the national soil census, which is far from the data requirements. At the same time, reclamation and plowing will change the nature of the soil, so it is impractical to directly use this method. The actual measurement includes the standard battery measurement method and physical and chemical properties measurement with the highest accuracy, but time-consumption, cumbersome procedures, equipment limitations, rainfall, and other unforeseen factors are drawbacks.

**Figure 3:**
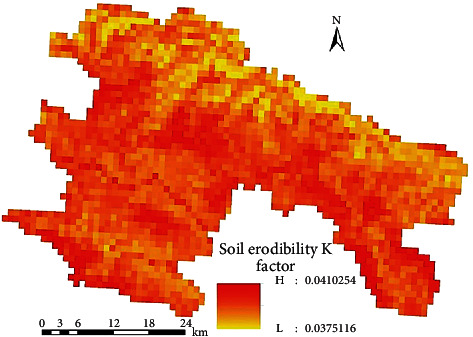
Spatial distribution of the soil erodibility *K* factor in a certain area.

The spatial distribution of the soil erosion *K* factor in a certain area is shown in Figure 3.

The soil erosion factor of Zone A ranges from 0.0375116 to 0.0410254, with an average of 0.0392685. From the macrodistribution of the soil erosion *K* value in China's water erosion area, it can be seen that the soil erosion coefficient *K* in a certain area is 0.0484–0.0091, with an average value of 0.0292; compared with this value, the calculation result of the soil erosion rate coefficient *K* in circle A is slightly higher than the provincial average. The north and northeast are bordered by the Yuanjiang River, with large dry heat evaporation and little precipitation. The conditions of erosion (precipitation) are not perfect, resulting in small and uniform *K*.

#### 4.2.3. P Factor of Soil and Water Conservation Measures

Common soil and water protection measures include reducing the slope, transforming the subsoil, and building ponds and dams. These measures mainly control soil erosion by affecting speed and flow. Among them, reducing the slope can effectively reduce water flow, and the construction of ponds and dams can adjust and reduce the flow. These measures are effective in reducing soil erosion and promoting soil protection.

The calculation and verification of the soil conservation function of county A based on the InVEST model are shown in Table 2.

The current research on the *P* value is mainly based on different land use types; due to the different actual conditions in the study area, the *P* value of the same land use type in different areas is different. When calculating the *P* factor, it is necessary to make extensive reference to related disciplines or similar research topics and then combine actual research to determine it. Due to its particularity, the *P* factor has a high degree of subjective flexibility when assigning values, so it is considered to be the most difficult to determine in the InVEST model. Area *A* is a mountainous area in southwest China, with a lot of deep and steep slopes, heavy rainfall, and excessive soil erosion. Soil protection measures such as afforestation, terrace construction, contour farming, and improvement of irrigation and drainage systems are adopted locally.

### 4.3. Evaluation and Characteristics of Soil Organic Matter

#### 4.3.1. Descriptive Statistical Characteristics of the Spatial Variability of Soil Nutrients at Different Scales

The statistical characteristics of soil available nutrients at different sampling scales are shown in Table 3.

It can be seen from Table 3 that on the sampling scale, AP has the highest change intensity, followed by AK, and AN has the lowest change intensity; on the field scale, AP shows the largest change intensity, followed by AN, and AK shows the smallest change intensity; on the plot scale, AN has the largest change intensity, followed by AP, and AK has the smallest change intensity.

#### 4.3.2. Spatial Autocorrelation Characteristics of the Spatial Variability of Soil Nutrients at Different Scales

The model parameters of the semivariance function soil nutrient index are shown in Table 4.

At the county level, the AN, AP, and AK regions are the largest; at the field scale, the range is reduced by 118.31 m, 55.41 m, and 112.21 m, respectively; at the block scale, the range further drops to 18.61 m, 43.31 m, and 27.61 m, leading to drastic changes in small-scale soils AN, AP, and AK and significantly reducing their scope.

#### 4.3.3. Scale Effect of Spatial Variability of Soil Nutrients

The fragmentation degree of the soil available nutrient map at different sampling scales is shown in Table 5.

The maximum effective phosphorus PD value is 1.18, and the distribution is relatively scattered. The main reason is that AP is more susceptible to random factors, which makes AP distributions disjoint, and has obvious local characteristics.

**Figure 4:**
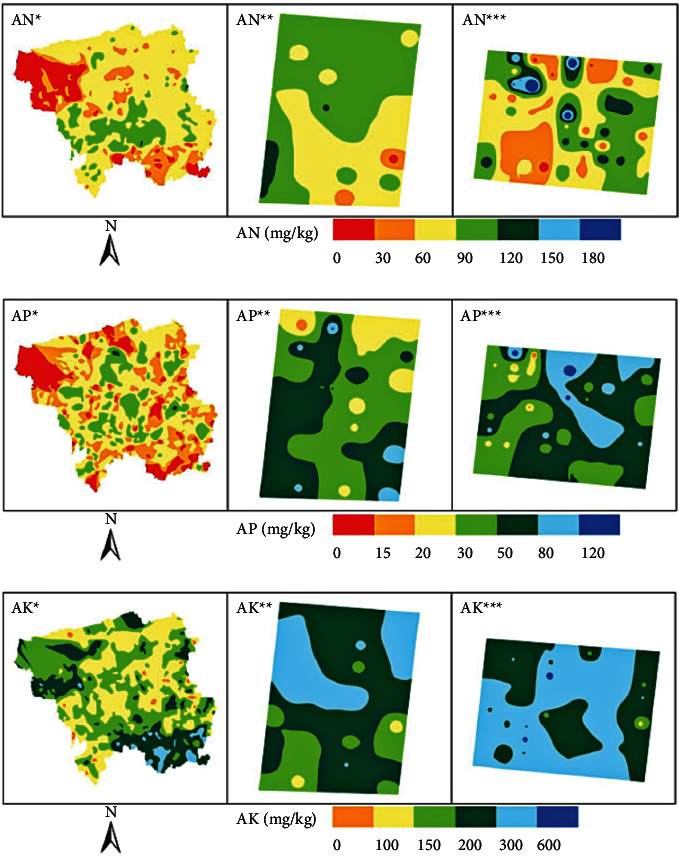
The spatial distribution of available nutrients at different sampling scales. (a) At the AN sampling scale of 0–180. (b) At the AP sampling scale of 0–120. (c) At the AK sampling scale of 0–600.

The spatial distribution of available nutrients at different sampling scales is shown in Figure 4.

It can be seen from the spatial distribution of nutrients that at the county level, except for a small part that is in a barren state, the alkali hydrolyzed nitrogen is continuously distributed in the soil, and most of the rest are at an average level.

**Figure 5:**
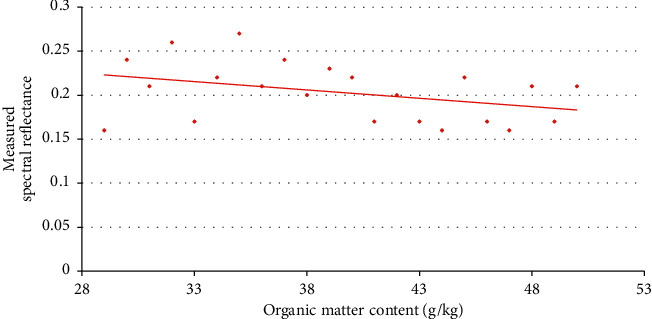
Fitting curve of soil organic matter content and measured sample point reflectivity on November 2, 2020. *y* = −0.0002*x*2 + 0.0019*x* + 0.2098 (fitness:0.835).

The corresponding curve diagram of the measured sample point of the soil organic matter content measured at the sampling point on November 2, 2020, is shown in Figure 5.

It can be seen from the obtained data that this paper adopts the method of polynomial modeling, combined with comparative experiments for research, selects one of the sample points of polynomial fitting, and uses the sample point as the control point.

**Figure 6:**
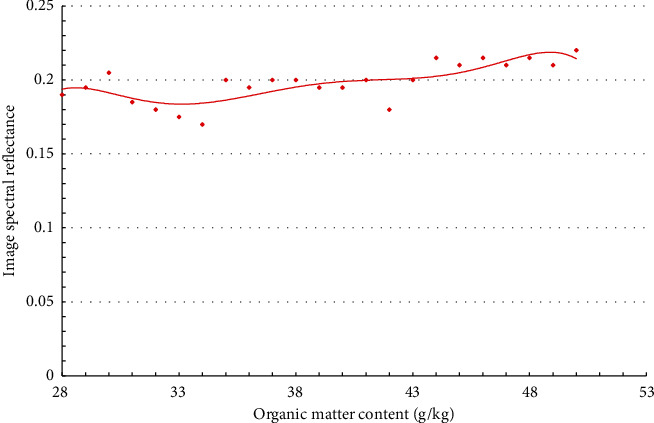
Fitting curve of soil organic matter content and image sample point reflectivity on November 2, 2020. *y* = 5*E* − 0.5*x*^3^ + 0.0017*x*^2^ − 0.0163*x* + 0.2202 (fitness:0.806).

The adjustment curve of soil organic matter content and reflectance at the image sampling point on November 2, 2020, is shown in Figure 6.

We select one of the sample points for polynomial fitting and check the sampling points, and the accuracy is similar, which proves that the model is very accurate and can be used to invert the soil organic matter in the entire area.

The statistical comparison table of the percentage of organic matter in a certain area in the three periods is given in Table 6.

Table 6 shows the distribution of organic matter content in the test area during the three time periods. The results show that the results of remote sensing inversion of organic matter are consistent with those of the organic matter content measured by field sampling. After land consolidation, the organic matter content in the experimental area is mainly distributed in the plains and above.

The statistical comparison table of the percentage of organic matter in a certain area in the three periods is given in Table 7.

Statistics for the distribution of organic matter over the three time periods in the study area show that the remote sensing inversion results of organic matter in the defined test area are consistent with the organic matter data obtained by field sampling. It can be seen from Table 7 that after land development and consolidation in a certain experimental area, soil fertility has been greatly improved, soil quality has been significantly improved, and the effect of land development and consolidation has been significant.

#### 4.3.4. BP Neural Network Inversion Model

The BP neural network is composed of input layer, hidden layer, and output layer.

The cumulative credibility of the first six principal components is shown in Table 8.

The 26 samples in a certain experimental area are divided into a prediction set and a verification set. There are 15 sets of predictions and 9 sets of verifications. The spectral band of the sample is calculated using the full spectrum, which requires a large amount of calculation and has nothing to do with the composition of the sample. The principal component analysis method can fully reflect the information of the original multiwavelength variables and perform principal component analysis of the prediction sample and the verification set.

The statistical comparison table of organic matter content in a certain area in two periods is shown in Table 9.

The inversion results in Table 9 are approximately equal to the inversion results of the polynomial inversion model. The results showed that the content of soil organic matter in this area increased significantly after land consolidation, which significantly improved soil fertility and quality.

### 4.4. Research on the Analysis Method of Soil Nutrient Variability in Farmland

Research in recent years has shown that the method of combining multisource satellite images and multitemporal images with laboratory spectral analysis and ground-based in situ research is the development trend of soil organic matter measurement.

Remote sensing is obviously the preferred method for estimating the surface temperature, because the main feature of remote sensing technology is that it can provide a wide range of data and images and use satellite data to calculate the surface temperature. Sensor technology is the theory and method for detecting satellite hot air duct data. At present, the remote sensing algorithms for surface temperature mainly include single-channel and multichannel satellite sensor infrared channel methods, infrared thermometer to measure surface temperature, and microwave remote sensing to monitor the surface temperature. At present, the use of infrared thermal data to monitor surface temperature is widely higher level.

Soil surface evapotranspiration is an important part of both surface heat balance and ecosystem water balance. The water balance determines the quantity and spatial distribution of water resources, and the heat balance between water and soil is very large. It determines the weather and climate change, which in turn determines the soil quality and the formation and development of ecosystems. Remote sensing technology is currently used to monitor surface evapotranspiration. There are mainly single-layer models and double-layer models, especially the SEBAL model based on the principle of energy balance equation. Its outstanding advantage is that it has a strong physical foundation and is suitable for different climates. The vegetation index is a simple and effective indicator to characterize the vegetation coverage and plant growth status. In recent years, with the rapid development of remote sensing technology, the use of remote sensing technology to extract the vegetation index has been relatively expanded. In the field of ecology, the vegetation index examines ecosystems on a global scale, and the results of patch-level ecosystem research are extended to the global space.

## 5. Conclusion

A wireless sensor network consists of sensor nodes with radio functions and is a very promising solution in the era of the Internet of Things. Although remote sensing technology eliminates the drawbacks of using it, there are still energy and equipment problems. Therefore, it is necessary to find new ways to solve the problems of energy and remote sensing technology. Through the development of related algorithms, this paper mainly solves the problems of power consumption, interference, intercluster communication, and channel stability in wireless sensor networks. The soil conservation module calculates and analyzes the functional strength of soil protection in a certain area in 2020, obtains the spatial distribution of soil environmental resistance, and compares the obtained results with the coefficient of determination to test the accuracy of the research. In terms of quantitative statistics, this article uses a combination of classical statistics and geostatistics, in addition to a geostatistical analysis model of remote sensing technology to examine specific areas. Obviously, after land consolidation, the fertility of the soil in the rectified area has increased, the content of related organic matter has also increased significantly, and the fertility and quality of the soil have been improved.

## Figures and Tables

**Table 1:** Information on latitude and longitude and rainfall erosivity *R* value of a certain area and its 6 surrounding counties and cities.

Area	Longitude (*E*)	Dimensions (*N*)	*R* factor value
Area A	102.824354	23.225596	4834
Area B	102.380196	23.367038	4094
Area C	102.823447	23.631394	3944
Area D	103.234147	22.787252	8784
Area E	102.403306	22.995532	7806
Area F	103.156728	23.377792	4987

**Table 2:** Calculation and verification of the soil conservation function in a certain area and county based on the InVEST model.

Land use type	*C* value	*P* value
Paddy field	0.06	0.06
Dry land	0.05	0.31
Woodland	0.03	0.99
Bush	0.03	0.99
Other woodlands	0.02	0.99
High coverage grassland	0.05	0.99
Low coverage grassland	0.18	0.99
Canal	0.15	0.01
Reservoir pond	0.21	0.01
Beach	0.02	0.06
Urban land	0.08	0.01
Rural settlement	0.07	0.01
Other construction lands	0.06	0.01

**Table 3:** Statistical characteristics of soil available nutrients at different sampling scales.

Scale	Variable	Min (mg·kg^−1^)	Max (mg·kg^−1^)	Mean (mg·kg^−1^)	SD (mg·kg^−1^)	Ske	Kur	sig	Coefficient of variation (%)	Distribution type
County	AN	9.01	169.01	80.83	21.33	0.44	0.83	0.07	26.37	N
	AP	1.01	93.01	23.08	14.21	1.38	2.53	0.04	61.48	LN
	AK	24.01	553.01	177.35	81.76	1.27	2.12	0.12	46.11	LN

Field	AN	16.83	126.88	56.12	24.61	0.88	1.03	0.66	43.85	N
	AP	19.58	133.86	55.44	29.08	1 .05	0.37	0.06	52.42	N
	AK	134.08	373.17	252.85	70.59	−0.13	−1.28	0.32	27.93	N

Plot	AN	15.28	264.47	88.14	47.72	1.06	1.58	0.37	54.12	N
	AP	15.33	153.44	63.38	29.23	0.75	0.34	0.63	46.12	N
	AK	170.12	669.83	308.87	80.42	0.56	2.86	0.17	26.04	LN

**Table 4:** Parameters of the semivariance function model of the soil nutrient index.

Scale	Variable	Theoretical model	Nugget*C*_*o*_	Sill*C*_*o*_ + *C*	Nugget/Sill (%)	Range (*m*)	Decisive factor *R*^2^	Residual (RSS)
County	AN	Exponential	0.00235	0.01549	15.13	410.01	0.429	9.73 × 10^−6^
	AP	Exponential	0.00891	0.07851	11.33	320.01	0.611	5.68 × 10^−5^
	AK	Exponential	0.00582	0.03245	17.92	390.01	0.638	1.96 × 10^−5^

Field	AN	Gaussian	0.00011	0.11821	0.09	118.31	0.424	6.16 × 10^−4^
	AP	Exponential	0.00011	0.04061	0.26	55.41	0.958	2.41 × 10^−7^
	AK	Gaussian	0.00002	0.01573	0.07	112.21	0.558	7.57 × 10^−6^

Plot	AN	Exponential	0.00241	0.05861	4.11	18.61	0.26	3.75 × 10^−5^
	AP	Exponential	0.00351	0.04631	7.57	43.31	0.731	1.58 × 10^−5^
	AK	Exponential	0.00154	0.01107	13.84	27.61	0.362	8.15 × 10^−7^

**Table 5:** Fragmentation degree of soil available nutrient map at different sampling scales.

Scale	Variable	Number of plaques NP (pieces)	Plaque density PD (pcs:hm^−2^)
County	AN	75	0.16
	AP	116	0.22
	AK	89	0.17

Field	AN	13	0.23
	AP	18	0.36
	AK	14	0.25

Plot	AN	48	1.75
	AP	33	1.18
	AK	18	0.71

**Table 6:** Comparison table of the percentage of organic matter content in a certain area in three periods.

Organic matter content (%)/time	<10	[10–20)	[20–30)	[30–40]	>40
July 17, 2014	16.523	32.592	31.087	8.958	10.841
November 2, 2019	3.407	6.554	14.056	30.072	45.912
June 16, 2020	6.666	13.304	10.011	40.262	29.756

**Table 7:** Comparison table of the percentage of organic matter content in a certain area in three periods.

Organic matter content (%)/time	<10	[10–20)	[20–30)	[30–40]	>40
July 2, 2003	76.527	10.592	6.82	4.924	1.123
November 27, 2019	37.407	46.552	8.967	4.715	2.356
June 7, 2020	60.666	17.334	13.011	5.911	3.075

**Table 8:** Cumulative credibility of the first six principal components.

Main ingredient	Cumulative reliability of the prediction set (%)	Cumulative credibility of the validation set (%)
P01	99.427	99.961
P02	99.576	99.937
P03	99.424	99.566
P04	99.821	99.347
P05	99.967	99.522
P06	99.978	99.431

**Table 9:** Statistical comparison table of organic matter content in a certain area in two periods.

Organic matter content (%)/time	<10	[10–20)	[20–30)	[30–40]	>40
July 17, 2015	16.724	32.497	30.177	9.246	11.357
November 2, 2020	3.336	6.676	13.348	30.037	46.602

## Data Availability

The dataset can be accessed upon request to the corresponding author.
